# Ephrin receptor A2, the epithelial receptor for Epstein-Barr virus entry, is not available for efficient infection in human gastric organoids

**DOI:** 10.1371/journal.ppat.1009210

**Published:** 2021-02-17

**Authors:** Nina Wallaschek, Saskia Reuter, Sabrina Silkenat, Katharina Wolf, Carolin Niklas, Özge Kayisoglu, Carmen Aguilar, Armin Wiegering, Christoph-Thomas Germer, Stefan Kircher, Andreas Rosenwald, Claire Shannon-Lowe, Sina Bartfeld

**Affiliations:** 1 Research Centre for Infectious Diseases, Institute for Molecular Infection Biology, Julius Maximilian University of Wuerzburg, Wuerzburg, Germany; 2 Department of General, Visceral, Vascular and Paediatric Surgery, University Hospital of Wuerzburg, Wuerzburg, Germany; 3 Institute of Pathology, Julius Maximilian University of Wuerzburg and Comprehensive Cancer Center Mainfranken, Wuerzburg, Germany; 4 Institute of Immunology and Immunotherapy, University of Birmingham, Birmingham, United Kingdom; Tulane University School of Medicine, UNITED STATES

## Abstract

Epstein-Barr virus (EBV) is best known for infection of B cells, in which it usually establishes an asymptomatic lifelong infection, but is also associated with the development of multiple B cell lymphomas. EBV also infects epithelial cells and is associated with all cases of undifferentiated nasopharyngeal carcinoma (NPC). EBV is etiologically linked with at least 8% of gastric cancer (EBVaGC) that comprises a genetically and epigenetically distinct subset of GC. Although we have a very good understanding of B cell entry and lymphomagenesis, the sequence of events leading to EBVaGC remains poorly understood. Recently, ephrin receptor A2 (EPHA2) was proposed as the epithelial cell receptor on human cancer cell lines. Although we confirm some of these results, we demonstrate that EBV does not infect healthy adult stem cell-derived gastric organoids. In matched pairs of normal and cancer-derived organoids from the same patient, EBV only reproducibly infected the cancer organoids. While there was no clear pattern of differential expression between normal and cancer organoids for EPHA2 at the RNA and protein level, the subcellular location of the protein differed markedly. Confocal microscopy showed EPHA2 localization at the cell-cell junctions in primary cells, but not in cancer cell lines. Furthermore, histologic analysis of patient tissue revealed the absence of EBV in healthy epithelium and presence of EBV in epithelial cells from inflamed tissue. These data suggest that the EPHA2 receptor is not accessible to EBV on healthy gastric epithelial cells with intact cell-cell contacts, but either this or another, yet to be identified receptor may become accessible following cellular changes induced by inflammation or transformation, rendering changes in the cellular architecture an essential prerequisite to EBV infection.

## Author summary

Epstein-Barr virus (EBV) is associated with malignancies of lymphoid and epithelial cell lineages, including gastric cancer (GC). Although EBV is only associated with up to 10% of GC, this unique subset is genetically and epigenetically distinct from other forms of GC. However, the sequence of events leading to EBV-associated GC (EBVaGC) remains unclear.

Ephrin receptor A2 (EPHA2) was identified as a receptor for EBV entry into epithelial cancer cell lines, yet the physiological relevance of its role in infection of healthy gastric epithelium was not explored. Using human adult healthy stem cell-derived gastric organoids, microscopy showed the EPHA2 receptor was strictly localized to cell-cell junctions and therefore inaccessible to EBV, resulting in poor infection. In contrast, EPHA2 expression was not confined to cell-cell junctions in cancer-derived organoids, rendering it accessible to EBV. Correspondingly, these organoids were more readily infected. Although EBV was not detected in healthy gastric epithelial tissue, immunohistochemical analysis identified EBV in inflamed epithelium. These results suggest viral entry requires initial changes to the gastric epithelium, likely induced by inflammation, to expose the virus receptor and enable efficient infection.

## Introduction

Epstein-Barr virus (EBV) is a gammaherpesvirus that infects more than 90% of the world’s population. While the virus is particularly well-known to infect B cells, causing infectious mononucleosis, Burkitt and Hodgkin lymphoma [1], it also infects epithelial cells and is associated with nasopharyngeal carcinoma and gastric cancer (GC) [2].

EBV-associated gastric cancer (EBVaGC) represents 8–10% of all GC cases worldwide, accounting for up to 80,000 cases per year [3,4]. EBVaGC was classified as one of four molecularly defined subtypes of GC, characterized by excessive cellular genome hypermethylation, frequent presence of *PIK3CA* mutations, overexpression of PD-L1/-L2 and CDKN2A silencing [3]. A causal role of EBV in this particular subtype of GC is expected due to the presence and clonality of the latent EBV episome in every cancer cell but not in surrounding tissue [5–10]. However, the exact sequence of events leading to EBVaGC is still a conundrum. Specifically, it is unclear whether viral entry into healthy cells initiates the pathogenic changes, or whether host cell modifications must precede the infection.

Virus entry into the main target cells is mediated by independent mechanisms; B cell infection requires the initial binding of the viral glycoproteins gp350 and gp42 to the B cell surface CD21 and HLA class II respectively (reviewed in [11]), followed by fusion mediated by viral envelope glycoproteins gH/gL and gB. In contrast, epithelial cell entry does not require gp350 or gp42. Instead, binding and fusion is directly mediated by gH/gL and gB and requires several entry receptors expressed on the epithelial cell surface, most prominently the ephrin receptor A2 (EPHA2) [11–13]. As members of the superfamily of transmembrane receptor tyrosine kinases, EPH receptors mediate short-distance cell-cell communication between neighboring cells upon binding of their ephrin ligands. EPH receptor-ephrin signaling plays a pivotal role during development but also in other cellular processes like adult stem cells niches, synaptic plasticity or bone homeostasis (reviewed in [14]). In primary epithelial cells, the location of EPH receptors is highly organized and restricted to cell-cell contacts [15,16]. It is unclear whether the receptor, tightly engaged in the junctions under homeostasis, is available for infection in healthy epithelium. Experimental studies of such questions have, however, been hampered due to the lack of a suitable primary cell model. Since the virus is strictly species-specific, there is no animal model [17] and most of the available studies have been performed on cancer cell lines. However, cancer cell lines markedly differ from *in vivo* tissue because they have accumulated mutations which alter cell organization, cell-cell contacts and signaling pathways. Here we use gastric adult stem cell-derived organoids [18,19] to analyze EBV infection of primary epithelial cells. In this culture system, human tissue-resident adult stem cells are seeded in an extracellular matrix and supplied with a mixture of growth factors. Stem cells proliferate and daughter cells differentiate to form 3-dimensional (3D) cell cultures, the organoids. To date, organoids can be grown from a vast variety of organs including the small intestine, liver, brain, prostate and stomach–each resembling the primary tissue they are derived from. They allow the study of a range of *in vivo* biological processes, including studies on viral and bacterial infection [20]. Of note, organoids have allowed the study of previously unculturable norovirus [21,22].

Our results support a role for EPHA2 in EBV entry into conventional cancer cell lines, as demonstrated previously. In contrast, organoids from normal, non-transformed tissue, are protected from viral infection, despite the expression of EPHA2. Subcellular localization analysis suggests that in normal organoids, EPHA2 is restricted to the cell-cell junctions which likely renders the protein inaccessible for the virus. Taken together, our results support the theory that cellular changes, probably in the cell architecture, are one prerequisite for infection.

## Results

### Upregulated EPHA2 expression results in more efficient EBV infection in epithelial cell lines

**Fig 1:**
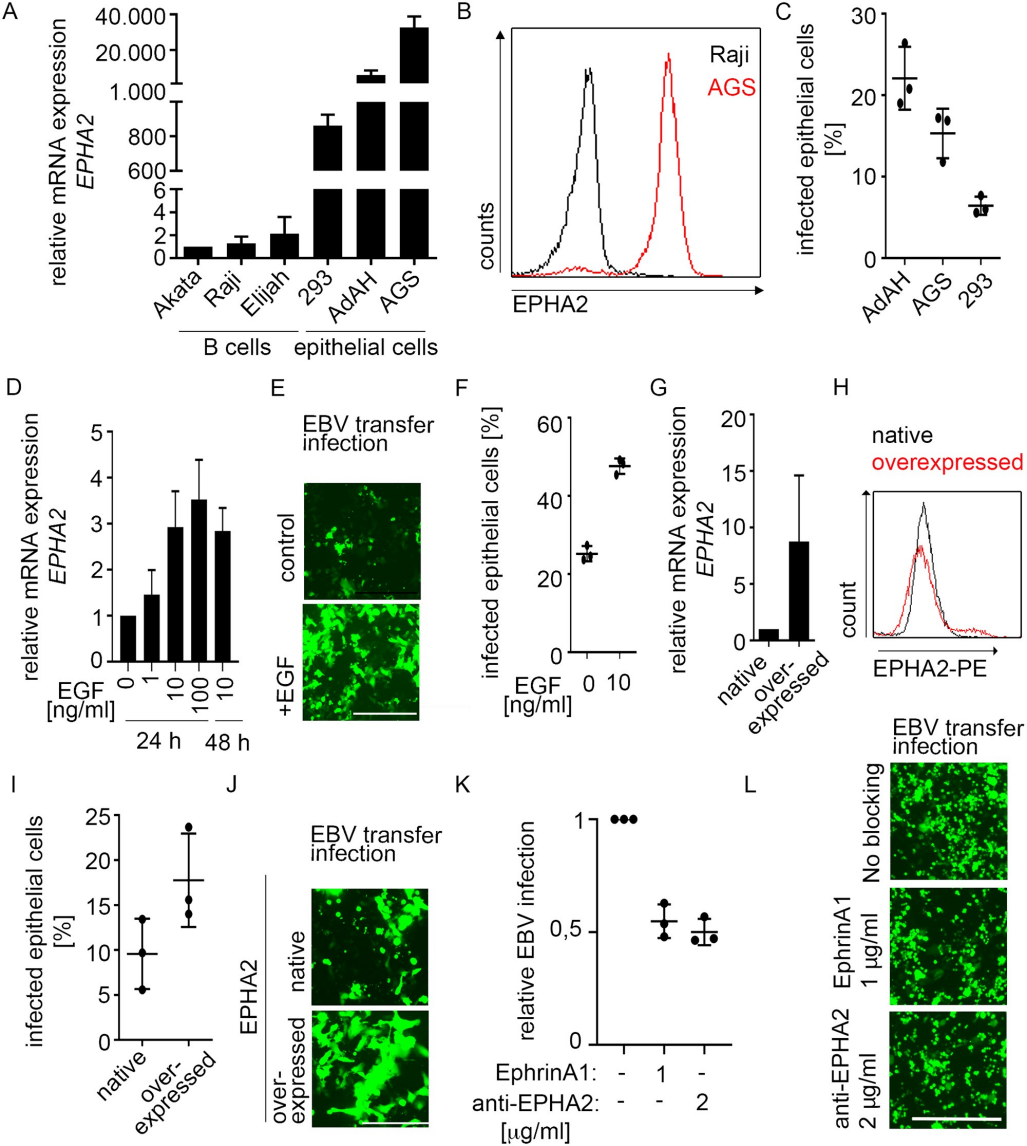
Increased EPHA2 expression leads to higher EBV infection efficiency in cell lines. (A and B) *EPHA2* mRNA was measured by RT-qPCR (A) and protein quantified by flow cytometry (B). (C) At 4 dpi, EBV transfer-infected cells were stained with CD45-APC antibody and analyzed by flow cytometry. (D-J) After EGF treatment or lentiviral overexpression of EPHA2 in AdAH cells, *EPHA2* expression was measured by RT-qPCR (D and G) or flow cytometry (H) and EBV transfer-infection efficiency was evaluated by fluorescence microscopy (E and J) and flow cytometry (F and I). (K and L) AdAH cells were incubated with EPHA2 ligand ephrinA1 or anti-EPHA2 antibody, infected by transfer infection and infected epithelial cells were measured by flow cytometry (K) and fluorescence microscopy (L). (A), (C), (D), (F), (G), (I) and (K) represent means with SD from three independent experiments. RT-qPCR results in (A), (D) and (G) were normalized to *GAPDH* expression and then to Akata B cells, sample without EGF or native cells, respectively. (E), (J), (L) show representative images from three independent experiments. Scale in E and L: 400 μm. Scale in J: 200 μm.

Recent reports demonstrated the importance of EPHA2 expression for EBV infection in conventional cancer cell lines [12,13]. To enable a comparison of infection in organoids and cell lines, we first validated the infection in cell lines. Cell lines derived from the lymphocyte lineage—Akata, Raji and Elijah—hardly expressed *EPHA2*, whereas three epithelial cell lines tested– 293, AdAH and AGS–highly expressed the receptor on RNA and protein level (Fig 1A and 1B), confirming published data [12]. Using B cell-mediated transfer infection with Akata B cells containing a GFP-expressing EBV [23], the infection efficiency of the cell lines ranged from 6–26%, with highest infection rates in AdAH cells (Fig 1C). *EPHA2* expression increased approximately three-fold after addition of 10 ng/ml epidermal growth factor (EGF) for 24 h and could not be further enhanced with excess of EGF (Fig 1D). The percentage of EBV-infected epithelial cells was doubled in EGF-pretreated AdAH cells compared to control cells (Fig 1E and 1F), corroborating previous results [13]. EPHA2 overexpression (Fig 1G and 1H) resulted in ~two-fold higher EBV infection efficiency of AdAH cells (Fig 1I and 1J). Blocking of EPHA2 receptor either with EPHA2 ligand or an antibody reduced, but did not abolish the infection (Fig 1K and 1L).

### EPHA2 is expressed heterogeneously in primary gastric epithelial cells

**Fig 2:**
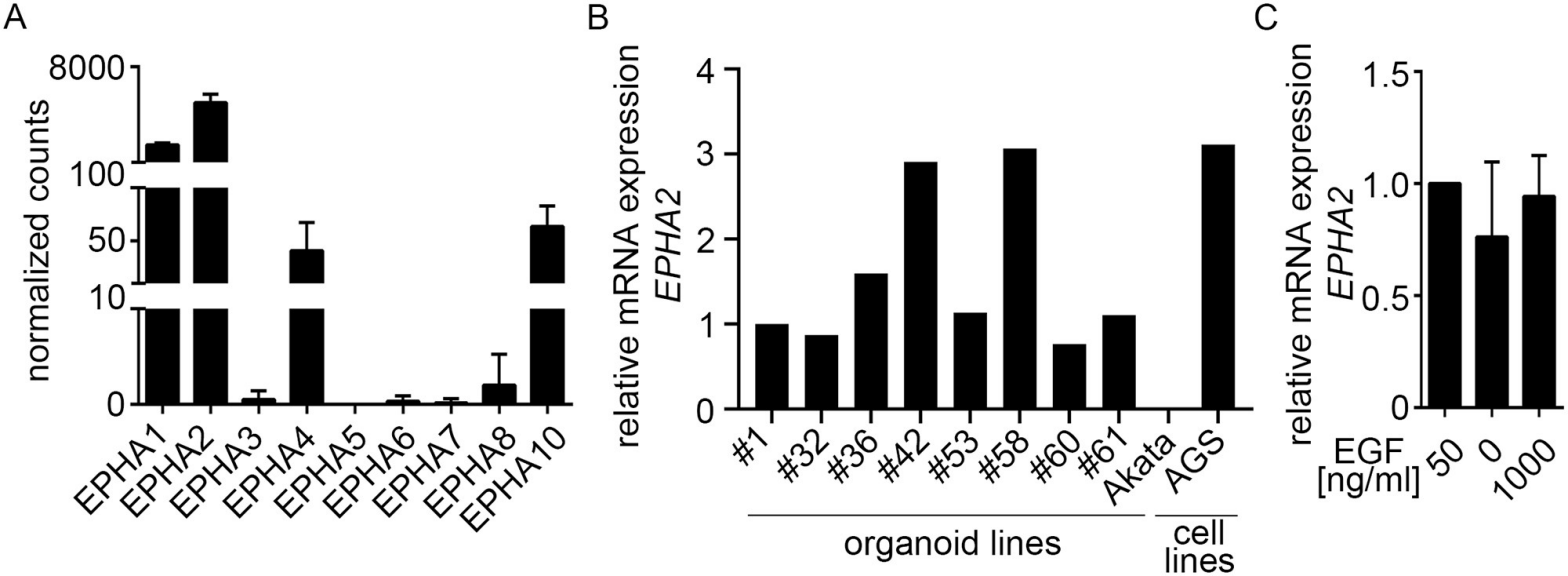
*EPHA2* is highly expressed in human gastric organoids. (A) Normalized gene counts of *EPHA* receptors are presented as means of six independent patient-derived organoids with SD. Data were obtained from total RNA-sequencing analysis, n = 3. (B) *EPHA2* expression of different patient-derived organoid lines was measured by RT-qPCR. Results were normalized to *GAPDH* expression and then to #1 organoids. #1–61 refers to patient IDs. (C) After EGF treatment of organoids, *EPHA2* expression was measured by RT-qPCR. Results represent means with SD from three independent experiments. Results were normalized to *GAPDH* expression and then to 50 ng/ml EGF.

To evaluate, whether EPHA2 could also allow infection of EBV into primary cells, we examined expression of EPHA2 in organoids. Total RNA sequencing of six human gastric organoid lines [24] showed prominent expression of *EPHA2* and other EPHA receptors (Fig 2A). RT-qPCR of several different organoid lines demonstrated that *EPHA2* expression levels varied in a patient-dependent manner but overall resided in the same range as for epithelial cell lines (Fig 2B). Organoid cultures grow under culture conditions that require 50 ng/ml EGF in the medium. Addition of higher EGF concentrations did not influence *EPHA2* expression (Fig 2C). Taken together, *EPHA2* is expressed in primary gastric epithelial cells at a comparable level as in cell lines.

### EPHA2 expression is not sufficient for EBV infection of human gastric organoids

**Fig 3:**
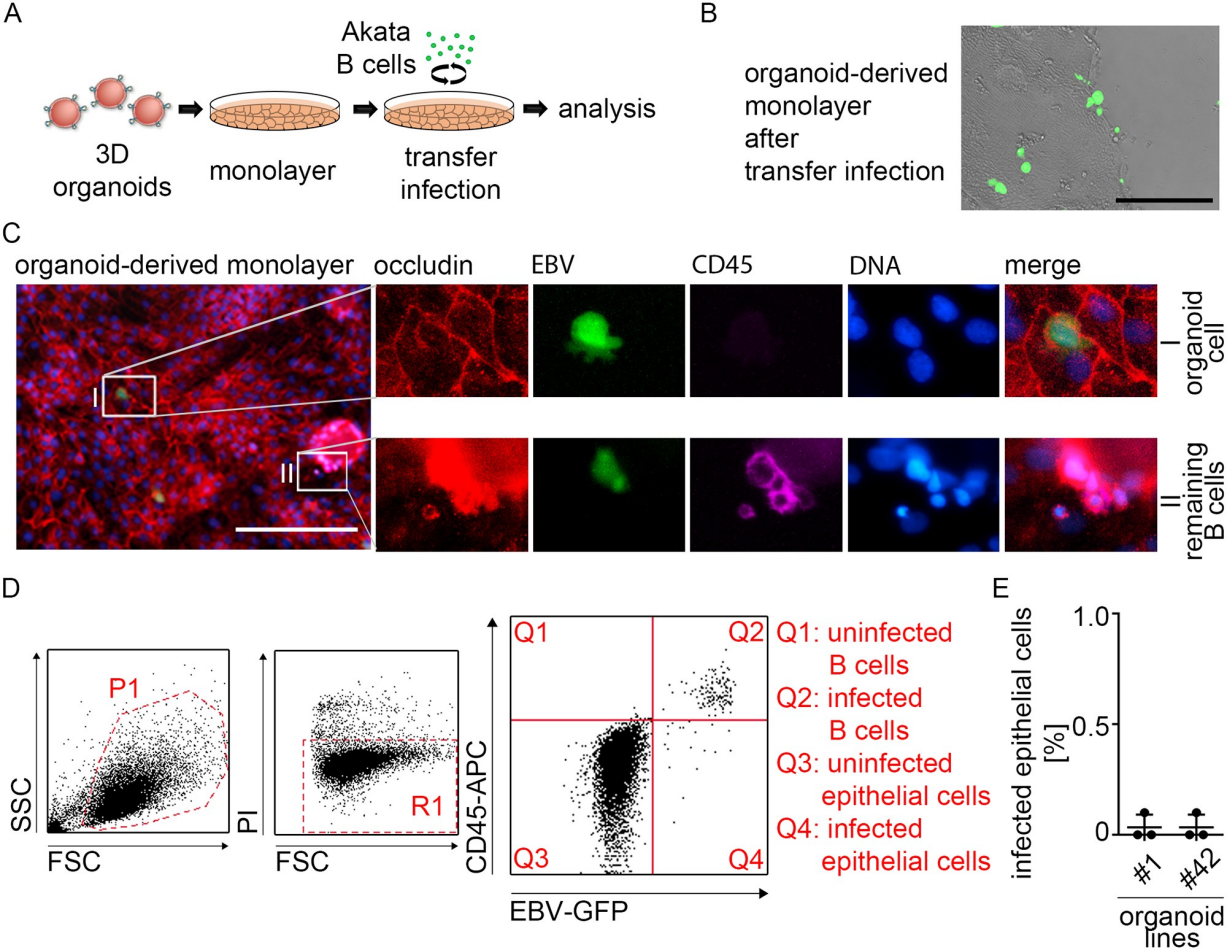
Despite comparable EPHA2 expression levels in organoids vs. epithelial cell lines there is no efficient EBV infection in human gastric organoids. (A) Scheme depicting B cell-mediated transfer infection of organoid-derived monolayers. (B) EBV transfer-infected organoid-derived monolayers were checked at 6 dpi by fluorescence microscopy. Scale: 200 μm. Representative image of at least three independent experiments. (C) At 6 dpi immunofluorescence was performed on EBV transfer-infected organoid-derived monolayers for epithelial marker Occludin, GFP-expressing EBV and lymphocyte marker CD45. DNA was counterstained with Hoechst. (I) depicts close-up of infected primary epithelial/organoid cell (GFP+, Occludin+ and CD45-). (II) depicts close-up of infected remaining B cells (GFP+, CD45+). Scale: 200 μm. Representative images of three independent experiments. (D) Flow cytometry gating strategy for evaluation of EBV infection efficiency. Left plot depicts FSC/SSC with gated cell population in P1. Middle plot depicts FSC/PI with gated viable cells in R1. Right plot depicts CD45-APC/EBV-GFP displaying localization of different cell populations. Q1: CD45+/GFP- = uninfected B cells, Q2: CD45+/GFP+ = infected B cells, Q3: CD45-/GFP- = uninfected epithelial cells and Q4: CD45-/GFP+ = infected epithelial cells. (E) At 4–6 dpi, EBV transfer-infected organoid-derived monolayers from different donors were analyzed for EBV infection rate by flow cytometry. Results are shown as means of three independent experiments with SD. #1 and 42 refers to patient IDs.

Since the epithelial entry receptor EPHA2 is expressed in organoids, we assessed EBV infection efficiency in cells from human gastric organoids. For infection, cells from organoids were seeded in monolayers and infected via B cell-mediated transfer as described above for AdAH cells (Fig 3A). At 4–6 dpi fluorescence microscopy displayed a number of GFP-positive cells (Fig 3B). However, control staining of CD45, a lymphocyte marker, showed that most GFP-positive (= EBV-positive) cells observed were remaining donor B cells (Fig 3C lower panel) and only single CD45-negative, GFP-positive epithelial cells could be detected (Fig 3C upper panel). To quantify EBV infection efficiency, we performed flow cytometry analysis from infected organoid-derived monolayers (Fig 3D). Staining with an anti-CD45 antibody confirmed that most GFP-positive cells were donor B cells (Quadrant 3) and less than 0.2% of cells could be detected in Quadrant 4, displaying newly infected epithelial cells (Fig 3E). Infection with cell-free virus yielded even lower numbers of infected cells (S1A Fig). To address the possibility that infection may be influenced by growth of the cells in 2D versus 3D, or infection via the apical versus the basolateral surface, we also performed microinjection of either cell-free virus, or donor B cells either to the apical or the basolateral side of the 3D organoids. Similar to that observed in 2D, neither parameter resulted in increased infection efficiency (S1B and S1C Fig). In summary, our data suggests that in organoids, expression of EPHA2 is not sufficient for EBV infection.

### Efficient EBV infection of human gastric cancer organoids

**Fig 4:**
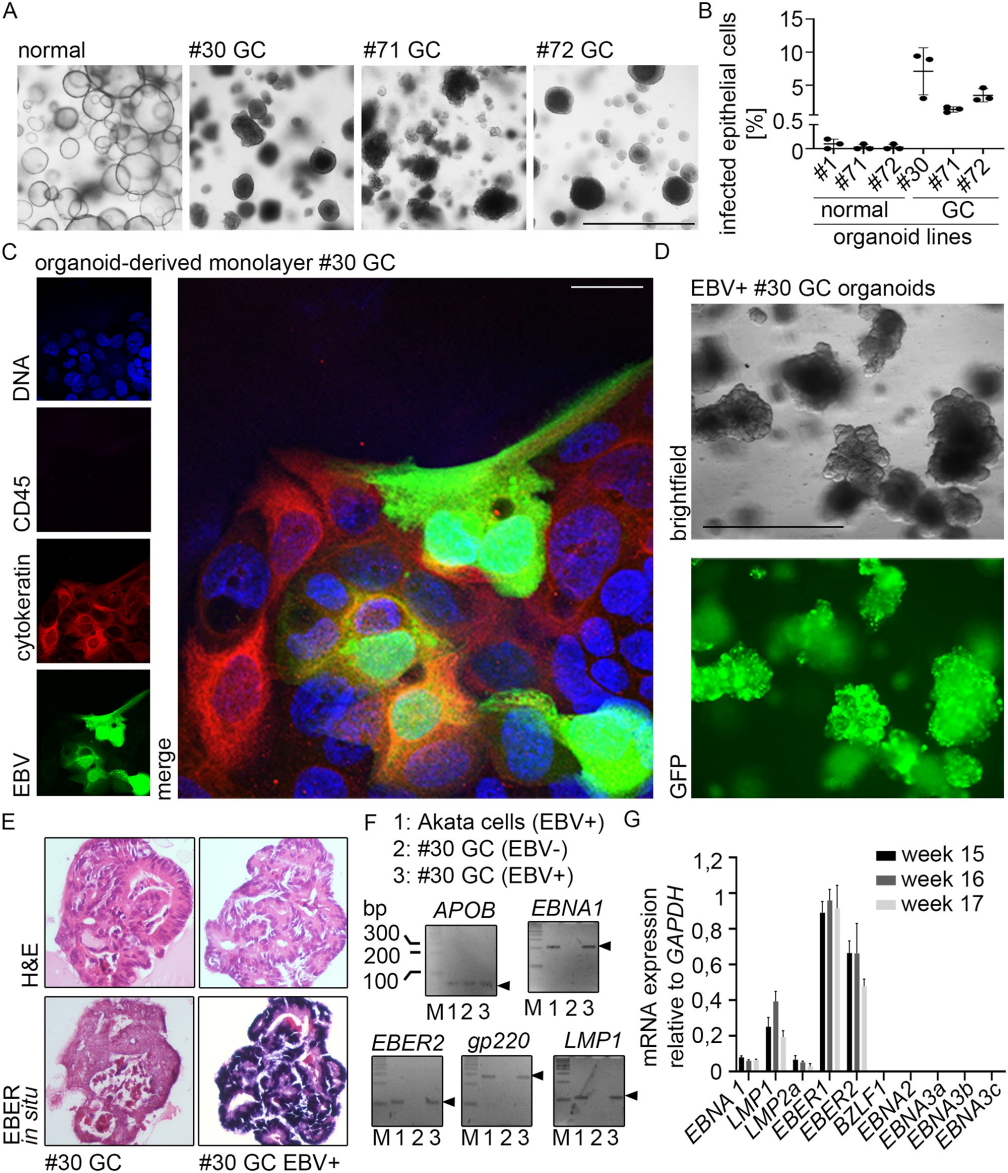
EBV can infect gastric cancer organoids. (A) Brightfield microscopy of normal and cancer organoids. #1–72 refers to patient IDs. Scale: 1000 μm. (B) At 6 dpi, EBV transfer-infected organoid-derived monolayers (normal and GC) were analyzed for EBV infection rate by flow cytometry. Results are shown as means of three independent experiments with SD. (C) At 4–6 dpi immunofluorescence was performed on EBV transfer-infected organoid-derived monolayers for epithelial marker Cytokeratin, GFP-expressing EBV and lymphocyte marker CD45. DNA was counterstained with Hoechst. Scale: 25 μm. Representative images of three independent experiments. (D) EBV transfer-infected #30 cancer organoid cells were FACS-sorted, clonally expanded and monitored by fluorescence microscopy. Scale: 1000 μm. (E) EBER in situ hybridization, detecting small non-coding RNA of EBV was performed on embedded clonal EBV+ or EBV- cancer organoids. (F) PCR analysis for the presence of EBV DNA (*EBER2*, *EBNA1*, *gp220* and *LMP1*) in clonal EBV+ or EBV- cancer organoids. *APOB* was used as eukaryotic control gene. (G) RT-qPCR was performed on RNA extracted from the infected #30 cancer organoid line. The viral gene expression profile included expression of *EBNA1*, *LMP1* and *LMP2a* plus the non-coding EBERs.

Organoids can also be generated from gastric cancer tissue, enabling paired culturing of normal and cancer organoids from the same patient [19]. Notably, cancer organoids also display the cellular diversity of the patients’ tumor [25] and biobanks of gastric cancer organoids contain all subtypes of cancers [26–28]. To explore the possibility to infect cancer organoids, we tested three EBV-negative cancer organoid lines (Fig 4A).

Flow cytometry quantification demonstrated 1–9% of infected cells in the cancer organoids and an up to 70-fold increase of infection comparing normal organoids and cancer organoids (Fig 4B). Immunofluorescence using control staining for CD45 to exclude remaining B-cells also confirmed infection in epithelial cells (Fig 4C). Cancer organoids were also susceptible to infection with cell-free virus, although the efficiency was lower than with B-cell transfer, as expected (S2 Fig). Sorted GFP/EBV-positive cancer organoid cells could be selected using a previously introduced neomycin resistance cassette in the EBV bacterial artificial chromosome [29]. After sort and 14 d expansion, cells were selected for 7 d in neomycin, picked and thereafter grown without selection pressure for over 6 months. In contrast to previous models using *ex vivo* cell lines or primary cells where the virus genome was lost over time [30–32], we obtained persistent infection (Fig 4D). EBER *in situ* hybridization initially confirmed the presence of the EBV small non-coding RNAs (EBERs) in this clonal line (Fig 4E). Conventional PCR was used to detect different EBV genes (Fig 4F). RT-qPCR confirmed EBV had established latent gene expression. This experimentally infected cancer organoid line exhibited mRNA expression of the virus maintenance protein *EBNA1* together with expression of the latent membrane proteins *LMP1* and *2a*. However, the cells did not express the viral *EBNA2*, *3a*, *3b* or *3c* mRNA, or indeed significant levels of mRNA of any viral lytic proteins, including *BZLF1* (Fig 4G). These data are consistent with the infected cells having a latency II phenotype. Taken together, in contrast to normal organoids, the cancer-derived organoids were able to maintain the viral genome and establish a long-term latent infection.

### Distinct localization pattern of EPHA2 in normal organoids versus cell lines and cancer organoids

**Fig 5:**
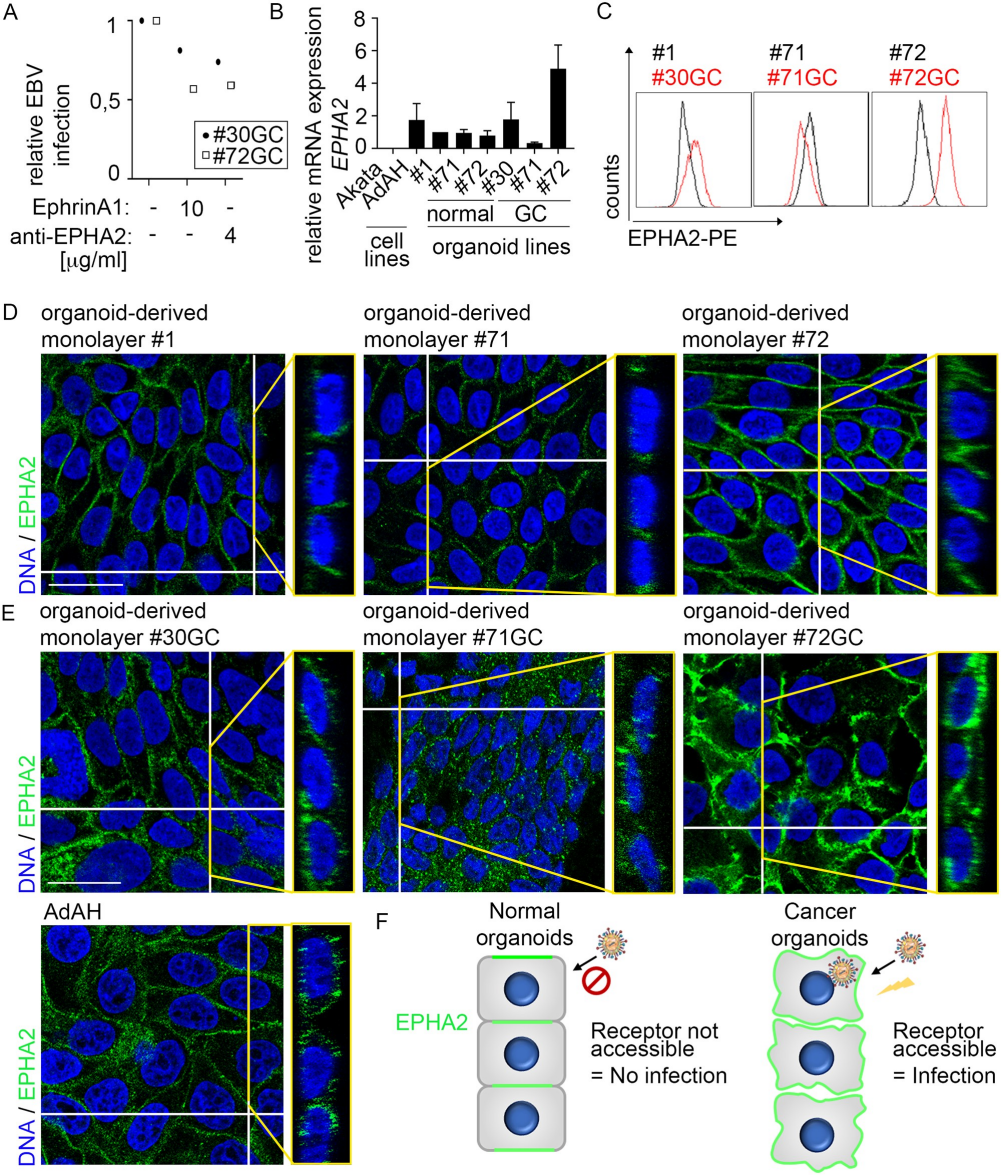
Localization of EPHA2 in GC organoid-derived monolayers resembles cancer cell lines and is different to normal organoid-derived monolayers. (A) Organoid-derived monolayers of two patients were incubated with EPHA2 ligand ephrinA1 or anti-EPHA2 antibody, infected by transfer infection and infected epithelial cells were measured by flow cytometry. (B) *EPHA2* expression of cell lines, normal organoids and GC organoids was measured by RT-qPCR. Results are shown as means with SD from three independent experiments. Results were normalized to *GAPDH* expression and then to the #1 normal organoids as control. #1–72 refers to patient IDs. (C) EPHA2 surface protein expression was measured by flow cytometry. Plots are representative of three independent experiments. (D) and (E) Immunofluorescence was performed for EPHA2. DNA was counterstained with Hoechst. Orthogonal view of the respective picture is depicted on the right. For separate channels, see S1 Fig Scale: 25 μm. (F) Scheme depicting localization and accessibility of EPHA2 for EBV entry in normal versus cancer human gastric organoid-derived monolayers.

Blocking of EPHA2 either by ligand ephrinA1 or anti-EPHA2 antibodies reduced the infection in cancer organoids similar to that observed in cancer cell lines (Fig 5A, compare with Fig 1K), supporting a role for EPHA2 in EBV infection. Therefore, to analyze the molecular mechanism of the observed differences in EBV infection efficiency between non-transformed and transformed cells, we turned again to EPHA2. The differences in the *EPHA2* mRNA expression and EPHA2 surface protein level were heterogeneous (Fig 5B and 5C). All normal organoid lines expressed about half of mRNA levels detected in the AdAH cell line. Cancer organoid lines expressed either similar, about 7-fold lower, or 2- to 3-fold higher mRNA levels as AdAH. We reasoned that if expression levels alone would influence infection, all cancer organoids would have higher expression. Because this was not the case, we searched for an alternative explanation.

Previous studies suggested that the localization of EPHA2 protein varies between epithelial cell lines and primary cells [15], pointing to the possibility that although expressed at similar levels, the protein may not be accessible for infection in primary cells compared to transformed cells. To test this hypothesis, we performed immunofluorescence (IF) analysis for EPHA2. In primary cells EPHA2 was located exclusively at sites of cell-cell contact (Fig 5D) and co-localized with the adherens junction marker E-cadherin but not apical actin filaments (S3 Fig). Similarly, in healthy tissue and 3D organoids EPHA2 was localized predominantly at the cell-cell junctions (S4 Fig).

In contrast, in cancer-derived organoids as well as cell lines, EPHA2 was not restricted to cell-cell contacts but also present at apical and basal sides of the cells, implying that this could possibly be an entry site (Fig 5E). These results support the hypothesis that if EPHA2 is an entry receptor, EBV might be unable to access it in non-transformed cells due to its sequestered localization (Fig 5F).

### Number of EBV-positive cells *in vivo* is dependent on inflammation status of epithelium

**Fig 6:**
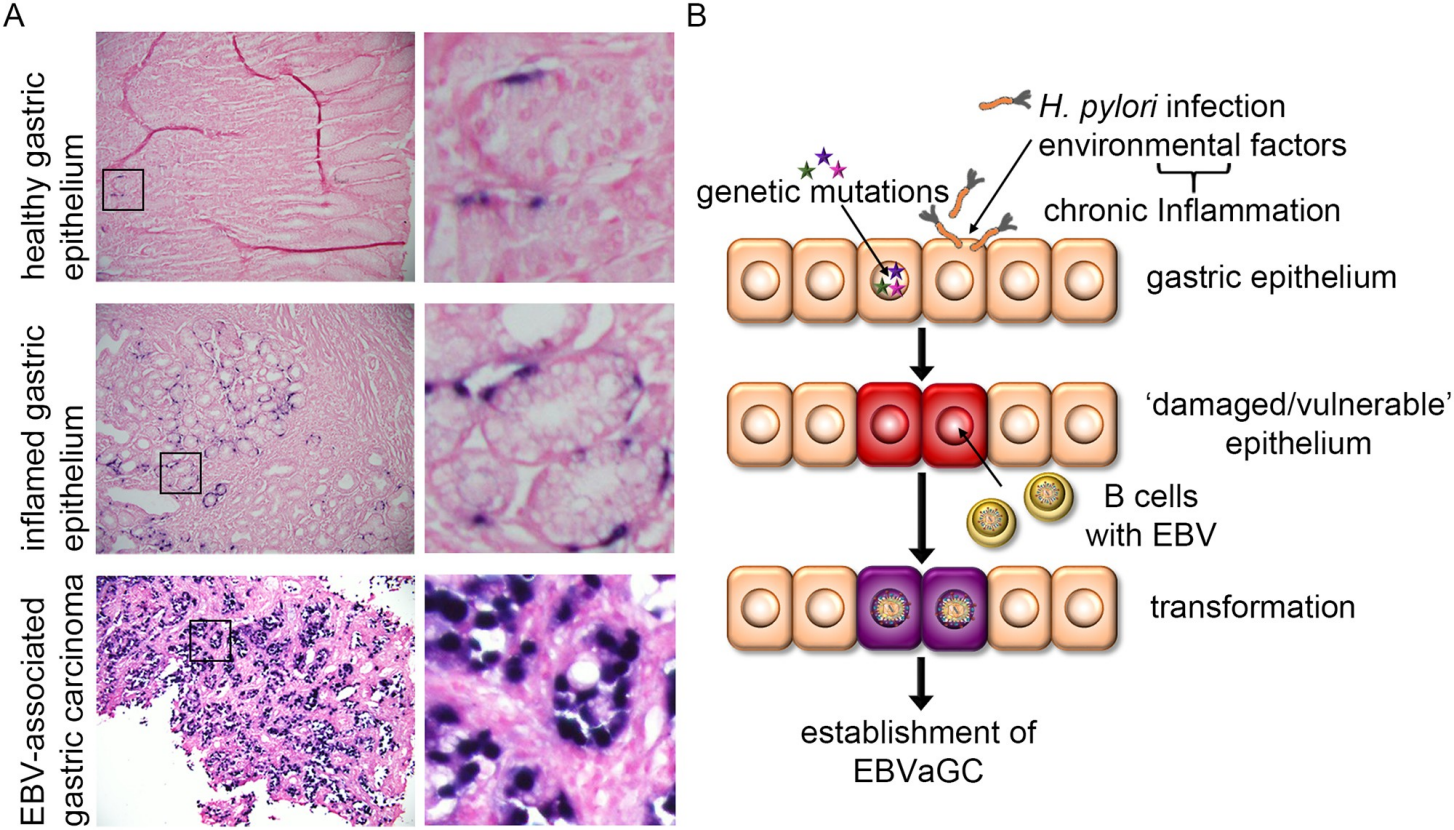
Efficient EBV infection requires “pre-damaged” epithelium. (A) EBER *in situ* hybridization was performed in embedded tissue detecting small non-coding RNA of EBV. Additional H&E staining was performed. Enlarged images on the right. (B) Working model for EBVaGC development. Genetic mutations and/or chronic inflammation, for example caused by chronic infection with the gastric pathogen *Helicobacter pylori (H*. *pylori)*, pre-damage the normal gastric epithelium, which thus allows for more efficient EBV infection mediated by infiltrating infected B cells and subsequent cell transformation resulting in carcinogenesis.

To evaluate EBV infection *in vivo* in gastric tissue, we performed EBER *in situ* hybridization. Results illustrated that hardly any EBV-positive cells could be found in healthy gastric epithelium. However, EBV-positive epithelial cells were abundantly present in inflamed gastric tissue. As expected and corroborating previous reports, in EBVaGC, every cell stained positive for EBV (Fig 6A). Taken together, we conclude that healthy gastric epithelium is unlikely to be infected with EBV, despite the presence of EPHA2. Our data suggests that prior changes in the epithelium could be necessary to render EPHA2 receptor accessible or to promote other cellular changes to allow EBV to infect primary gastric epithelial cells (Fig 6B).

## Discussion

EBVaGC accounts for over 8% of all GC cases [3]. The presence of a monoclonal virus genome in gastric epithelium from patients with GC as well as pre-malignant disease stages including chronic atrophic gastritis, but not healthy tissue [33], implies a causal role for EBV in the pathogenesis of gastric carcinoma. Yet, our understanding of how EBV initially accesses the gastric epithelium and establishes a persistent infection to drive the malignant changes in these cells remains poor.

In 2018, two independent papers determined EPHA2 as the EBV entry receptor in a variety of cancer cell lines including the gastric adenocarcinoma epithelial cell line AGS [12] and the nasopharyngeal carcinoma cell lines CNE1, CNE2 and HNE1 [13]. In these cells, EBV internalization and fusion was shown to be triggered by an interplay between EPHA2 and the viral entry glycoproteins gH/gL and gB. Interestingly, EPHA2 had likewise been demonstrated to function as an entry receptor into epithelial and endothelial cells for the Kaposi’s sarcoma-associated herpesvirus (KSHV), the second human γ-herpesvirus besides EBV [34]. Again, entry is mediated by the highly conserved herpesvirus glycoproteins gH/gL and their interaction triggers EPHA2 phosphorylation and endocytosis of KSHV [35–37].

Using a similar set of epithelial cell lines, we confirmed that increased EPHA2 expression resulted in increased EBV infection, and correspondingly decreased EPHA2 accessibility due to ligand binding or blocking antibodies to EPHA2 resulted in decreased EBV infection. The range of effects that we observed (about 50% reduction of infection by blocking) matched the effects seen with these specific reagents in the published experiments. More efficient reagents may further increase the observed effects as published [12,13].

Also, although previously published experiments used cell-free virus, we used infection mediated by transfer from lytic B cells. It is possible that the dependence on EPHA2 may be reduced when virus is directly transferred in this manner, implying transfer infection may require additional receptors, such as integrins [38]. This alludes to the physiological relevance of transfer infection of epithelial cells. EPHA2 receptor is upregulated by inflammatory cytokines [39] and has also been shown to play a role in the recruitment of leukocytes to the site of inflammation [39–41]. Similarly, certain integrins are activated by inflammatory cytokines and also aid the recruitment of leukocytes. This suggests EBV-infected B cells (or lytic plasma cells) may themselves be recruited to sites of inflammation, aided by EPHA2 whilst making use of its expression to enter the epithelial cells. Indeed, such B cells are readily found in regions of intestinal inflammation such as inflammatory bowel disease and ulcerative colitis [42,43]. It has also been suggested that chronic inflammation could render epithelial cells sensitive to EBV infection (reviewed in [44–46]). Correspondingly, we identified EBV-infected epithelial cells in inflamed gastric tissue. Together, our data supports a function for EPHA2, but also points to the requirement for another receptor(s), such as integrins or another, yet unidentified receptor.

Our results in organoids indicate that in healthy gastric epithelial cells EPHA2 is located predominantly within the cell-cell junctions. This suggests that other events must precede the infection to allow accessibility of EPHA2 such as chronic inflammation or tissue damage which is known to alter the localization/expression of cellular receptors. However, our results do highlight that EBV infection of healthy gastric epithelial cells is likely to be an extremely rare event, which is unlikely to result in the establishment of a persistent latent infection.

Generally, EPHA2 is highly expressed in epithelial cells [47]. The role of the EPH system in cancer is complex. Up- as well as down-regulation of EPH expression had been reported in the literature (reviewed in [48,49]). Because the results from cancer cell lines indicated that expression levels of EPHA2 influenced infection—a two-fold to five-fold increase of expression resulted in double the numbers of infected cells—we compared the expression levels in cancer cell lines and organoids. Cancer organoids were infected with strikingly higher efficiency than the normal lines. In line with this, EBER *in situ* hybridization studies showed a lack of EBV infection of non-malignant epithelial cells [6,48–50]. However, comparing cancer organoid lines from three patients, expression levels of EPHA2 did not fully mirror infectivity, and one cancer organoid line with very low expression level of EPHA2 was also infectable. This indicates, that additional factors other than mere expression of EPHA2 may be affecting EBV entry into epithelium.

One of the major differences between normal and transformed epithelium is the changes in cell-cell junctions triggered by epithelial to mesenchymal transition, whereby E-cadherin expression, amongst other junctional proteins, is downregulated resulting in a loss of polarity. Indeed, downregulation of E-cadherin has been observed in tumors of epithelial origin (reviewed in [6,50–52]), EBV-infected NPC [53], GC [54] and EBVaGC in particular [55]. E-cadherin and its associated complex dictates both the polarity and the motility of epithelial cells [56] and importantly, E-cadherin regulates the function and localization of EPHA2 [15,57]. Thus, a downregulation in E-cadherin expression might be expected to result in an altered localization of EPHA2. Indeed, EPHA2 exhibited a striking alteration in its localization on the cancer organoids and cell lines, exhibiting diffuse expression rather than being restricted to cell-cell junctions, as previously reported for transformed cells [15,57,58].

In contrast to the transformed cells, our normal organoids exhibited EPHA2 localization predominantly to cell-cell junctions [59,60] and EPHA2 co-localized with the prominent adherens junction marker E-cadherin [61,62], confirming earlier publications [15,16]. These results indicate that under physiologic conditions EBV infection of normal healthy epithelium may be limited by the inaccessibility of the entry receptor located within the cell-cell junctions. Other viruses such as adenovirus, coxsackie B virus or herpes simplex virus likewise use receptors in cell-cell junctions. The receptors were shown to be inaccessible to the virus unless junctions were disrupted (reviewed in [63]). Thus, we propose inflammation or pre-neoplastic changes as a prerequisite to render gastric epithelial cells susceptible to infection with EBV.

Taken together, we propose that the presence of EPHA2 on primary gastric epithelial cells in itself is not sufficient for efficient EBV infection. Other key drivers such as inflammation or somatic mutations likely changing the cell architecture are required to enable EBV infection, persistence and establishment of latency. The conceivable underlying mechanism might be a shift in the localization and hence accessibility of EPHA2 or another–yet to be identified—receptor. Subsequently, oncogenic hits induced by the virus could cooperate with previous events to accomplish malignant transformation towards EBVaGC.

## Materials and methods

A detailed description of further materials and methods can be found in the online S1 Methods.

### Ethics statement

Our study was reviewed by the ethical committee of the University Clinic, Wuerzburg, approval # 16/36. Human gastric tissue for the University of Birmingham study were obtained from the Human Biomaterials Resource Centre, Human Tissue Authority License number 12358, under the Research Tissue Bank ethical approval 20/NW/0001.

### Cell lines

B cell (EBV+ Akata [29], Raji [64,65] and Elijah [66,67]) and epithelial cell lines (AdAH [23], AGS (ATCC CRL 1739) and 293) were grown in RPMI 1640 (Sigma-Aldrich, R8758) supplemented with 10% fetal calf serum (FCS) (Merck Millipore, S0615/1109D) and 1% penicillin-streptomycin (P/S) (Thermo Fisher Scientific, 15140122). For lentivirus production, 293FT cells (Thermofisher Scientific, R70007) were cultured in DMEM high glucose (Gibco, B1966021) with 10% FCS, 1% P/S, 2 mM L-glutamine (Gibco, 25030081) and 1 X MEM non-essential aminoacid solution (Gibco, 11140050). EBV+ Akata cells contain the latent EBV bacterial artificial chromosome harboring a GFP and a neomycin resistance gene for selection purposes [29]. Akata cells as well as 293FT cells were selected with 167 μg/ml and 500 μg/ml G418 (Sigma-Aldrich, A1720), respectively. All cell lines were maintained in 5% CO_2_ at 37°C and were split twice a week.

### Patient-derived human gastric organoids from normal and cancer tissue

S1 Table contains a list of patient information. Human gastric organoids were generated from isolated gastric glands to be maintained in culture as described previously [19,28] and kept at 37°C, 5% CO^2^ in a humidified incubator. The medium (S2 Table) was changed every 2–3 d and organoids were passaged 1:8 every 7–14 d.

For 2D cultures, organoids were mechanically disrupted and seeded as pieces or single cells on conventional plastic (24- or 48-well plates) for infections or 8-well μ-slides for confocal microscopy (IBIDI, 80826). Single cell suspension was generated by treating mechanically disrupted organoids for 10 min with TrypLE Express (Gibco, 12605028) at 37°C.

### EBV B cell-mediated transfer infection

For B cell-mediated transfer infection, EBV+ Akata cells [29] were induced to activate the virus lytic cycle using 10 mg/ml human immunoglobulin (IgG) (MP Biomedicals, 0855049) for three days. In standard assays, 10^*6*^ donor cells (induced Akata) were added to a ~ 80%-confluent well of a 24-well plate that had been seeded 24 h before with 0.5–3.0 × 10^*5*^ acceptor cells in the case of cell lines. Organoids seeded in 2D monolayers were usually cultured for 7–14 d until near-confluency before EBV transfer infection. After co-culturing for 24 h, donor cells were removed from acceptor cells by vigorous washing; transfer infection rate was assayed 72–96 h after the initiation of co-culture via fluorescence microscopy (Evos FL Imaging system) and flow cytometry (BD Accuri^™^ C6 Flow Cytometer) via the incorporated green fluorescence protein (GFP) gene in the EBV BAC. To differentiate between donor B cells and acceptor epithelial cells, additional staining with an APC-labelled CD45 antibody (Invitrogen, MHCD4505) was performed. Propidium iodide (PI) (Sigma Aldrich, P4864) staining was used to exclude dead cells.

### Immunofluorescence (IF)

For IF of 3D organoids, organoids in Matrigel were resuspended in 500 μl cell recovery solution (Thermo Fisher Scientific, 12648–010) with a widened pipette, transferred to falcon tube and incubated for 45 min on ice until Matrigel was dissolved. For IF in 2D, dissociated gastric organoids were seeded onto 8 well μ-slides (IBIDI, 80826) to form 2D monolayers for immunofluorescence staining. Cells were grown for 7–14 d to reach approximately 90% confluency. Fixation was performed with 4% PFA for 20 min at room temperature, washed three times with PBS and permeabilized in 1 X PBS supplemented with 0.3% Triton-X, 1% DMSO and 1% fresh BSA for 1 h. Stainings were performed with primary antibodies (Occludin: Santa Cruz Biotechnology, sc-133256; CD45: Santa Cruz Biotechnology, sc-1178; EPHA2: Cell Signaling, 6997S; Pan-cytokeratin: Santa Cruz Biotechnology, sc-8018; E-cadherin: BD Bioscience, 610182) in 1 X PBS supplemented with 5% goat serum (Thermo Fisher Scientific, 31872) overnight at 4°C followed by Alexa Fluor (AF) 488 or AF647-conjugated secondary antibodies (Cell Signaling) in 1 X PBS supplemented with 5% goat serum for 3 h at room temperature. Actin filaments were stained with Phalloidin (Thermo Fisher Scientific, A22283) and DNA was stained with Hoechst 33342. After washing three times with PBS, stained cells were visualized using a confocal microscope (Leica, TC5 SP5 X).

### RT-qPCR for EBV gene expression

Absolute quantification of EBV gene transcripts by RT-qPCR was performed as previously described [68] and detailed in S1 Methods. All primer sequences are listed in S3 Table.

## Supporting information

S1 Methods A detailed description of further materials and methods. (PDF)

S1 Fig EBV infects healthy gastric epithelial cells very inefficiently. (A) Organoid-derived monolayers were infected with cell-free virus or by transfer infection. 1 dpi, EBV infection efficiency was evaluated by flow cytometry. Bars represent means with SD of 16 experiments in organoids derived from 6 patients. (B and C) Organoids were microinjected with EBV-positive, lytically induced Akata B cells or cell-free virus at the apical or basolateral side. (B) Illustration and representative image of microinjected organoids. (C) At 4 dpi, EBV infection efficiency was evaluated by flow cytometry. Data represent means with SD from two independent experiments.
(PDF)

S2 Fig B cell-mediated transfer infection more efficient than cell-free virus infection in cell lines as well as organoids. At 4 dpi, EBV infection efficiency was evaluated by flow cytometry (A) and fluorescence microscopy (B). (A) Data represent means with SD from two independent experiments. (B) Representative images from two independent experiments. Scale: 400 μm. #30 and 72 refer to patient IDs.
(PDF)

S3 Fig In normal but not cancer organoid-derived monolayers, EPHA2 co-localizes with E-cadherin in cell-cell junctions. (A) Immunofluorescence was performed for EPHA2 and E-cadherin. DNA was counterstained with Hoechst. Scale: 25 μm. (B) Co-localization analysis for EPHA2 and E-cadherin was performed using ImageJ. Mander’s coefficients M1 and M2 with SD were calculated from four individual images (1). (C) Scheme depicting localization of EPHA2 and E-cadherin in adherens junctions of normal human gastric organoids. AJ: adherens junction, TJ: tight junction. (D) Immunofluorescence was performed for EPHA2. DNA was counterstained with Hoechst. Scale: 25 μm. (E) Immunofluorescence was performed for EPHA2. Actin filaments were stained with Phalloidin, DNA was counterstained with Hoechst. #1, 30, 71, 72 refers to patient IDs. Scale: 25 μm. Images in A and D are identical with images shown in Fig 5C and 5D in the main manuscript. The separate display was chosen for space reasons: The main manuscript contains the overlay of EPHA2 and DNA and the supplement contains full display of separate channels.
(PDF)

S4 Fig EPHA2 localizes to cell-cell junctions in normal 3D organoids as well as in gastric tissue. (A) Immunofluorescence was performed for EPHA2 and cell-cell contact marker E-cadherin. DNA and actin filament counterstaining with Hoechst and phalloidin respectively indicate the orientation of the cells with the apical side facing the lumen of the organoid. Images were taken on a confocal microscope and the 3D reconstruction was built by LAS software (Leica). (B) Images of paraffin sections of healthy gastric mucosa or cancer tissue stained for EPHA2. Scale: (A) 20 μm, (B) 10 μm.
(PDF)

S1 Table Patient information for the organoid lines used in this study. (PDF)

S2 Table Organoid medium composition for human gastric organoids. ROCK inhibitor was added only after the initial seeding and passaging of the organoids. For basal medium (AD++), Advanced Dulbecco’s modified Eagle medium (DMEM)/F12 supplemented with 10 mmol/l HEPES and GlutaMAX 1 X was used. CM: conditioned medium; inh.: inhibitor; N-Ac: N-acetylcysteine; EGF: epidermal growth factor; FGF-10: fibroblast growth factor-10; TGF-β: transforming growth factor-β; ROCK: Rho-associated coiled-coil forming protein serine/threonine kinase.
(PDF)

S3 Table Primer sequences. (PDF)

S1 Data Original data: Excel file with values behind means and standard deviation used to build graphs. (XLSX)

S2 Data Original data: Image of the full gel shown in 4F. (JPG)
