# Supplementary material for: Ephrin receptor A2, the epithelial receptor for Epstein-Barr virus entry, is not available for efficient infection in human gastric organoids
Source: PLoS Pathog. 2021 Feb 17;17(2):e1009210. doi: 10.1371/journal.ppat.1009210 (PMC7935236; doi:10.1371/journal.ppat.1009210)
Supplement: S1 Methods — (PDF) [file ppat.1009210.s001.pdf]

SUPPLEMENTARY MATERIALS AND METHODS

EPHA2 overexpression plasmid

The full-length *EPHA2* complementary DNA (cDNA) fragment was cloned into a lentiviral expression plasmid (pLenti-puro, Addgene #39481) for EPHA2 overexpression experiments. The *EPHA2* cDNA sequence was amplified by PCR from the plasmid pCLXSN-EphA2-Flag (Addgene #102755) using Phusion High-Fidelity (HF) DNA polymerase (NEB, B0518S). Primer sequences are listed in supplementary table 3.

Lentivirus production, concentration and transduction

Lentivirus was produced by transient co-transfection of 293FT cells with a three-plasmid combination. Briefly, a 100 mm dish of ~70% confluent cells was transfected with 6 µg of psPAX2 (Addgene, 12260), 2 µg of pVSV-G (Addgene, 12259), and 4 µg of lentiviral expression plasmid mixed with 3 ml Opti-MEM (Thermo Fisher Scientific, 31985070) containing 36 µl Lipofectamine 2000 (Thermo Fisher Scientific, 18324012). 24 h post-transfection medium was replaced.

96 h post-transfection lentivirus was harvested via centrifugation at 600 x g for 5 min at RT. Subsequently, virus was sterile filtered (0.45 µm) and concentrated via ultracentrifugation at 19,500 rpm for 2 h at 16 °C (SW40Ti Swing Rotor in Beckman Coulter OptimaTM L-80 XP Ultracentrifuge). Afterwards, the virus pellet was resuspended in 250 µl cell maintenance medium depending on the respective target cells.

For AdAH cells, 50 µl of concentrated virus were used for infection of a 24-well that had been seeded at 0.5 x 10^5 cells/well the previous day. 1 ml of medium was added to the cells and incubated at 37 °C.

Flow cytometry analysis for protein expression

Surface protein expression was analyzed by flow cytometry (BD Accuri™ C6 Flow Cytometer). Cells were washed once with PBS + 10% FCS, then the fluorescently-labelled antibody was added (PE-coupled EPHA2 antibody (Biolegend, 356804)). The cell-antibody suspension was incubated for 30 min protected from light at 4 °C. After one washing step, cells were analyzed.

RT-qPCR

RT-qPCR was performed as published previously (2). Briefly, RNA was isolated from organoids according to the manufacturer's recommendations (RNeasy Mini kit; Qiagen). For RNA isolation from organoids, Matrigel was first removed from the cultures by gentle disruption with cold Advanced DMEM. Complementary DNA was generated using established reverse transcriptase protocols (M-MuLV, NEB). Quantitative reverse transcriptase PCR (RT-qPCR) was performed using SYBR green (Bio-Rad) and the CFX 384 Real Time system (Bio-Rad). Results were calculated using the ΔΔCt method. Relative quantification was achieved by normalizing results to the values obtained for glyceraldehyde-3-phosphate dehydrogenase (*GAPDH*). Primer sequences are listed in supplementary table 3 (S3 Table).

EBER *in situ* hybridization

EBER *in situ* hybridization was carried out in each sample on 5 µm-thick section using the Ventana BenchMark ULTRA automatic immunostainer following the manufacturer's recommendations (Ventana Medical Systems, Tucson, AZ, USA). A control slide was prepared from a paraffin-embedded tissue block.

RNA sequencing analysis

RNA-seq data were curated from GSE127938 (2).

EPHA2 staining gastric tissue

FFPE slides of tumour were deparaffinised in xylol for 25 min and subsequently rehydrated. Antigen retrieval was performed in 10 mM citric acid monohydrate buffer (pH 6.0) for 13 min under pressure and endogenous peroxidase blocked with 3% H_2O_2. Primary antibody (EPHA2

(D4A2) Cell Signaling Technology Systems, 1:400), was incubated for 1 h at RT and signal amplification was achieved by the Advance HRP Link Kit for 40 min and developed for 10 min with the DAB+ Liquid Kit (Dako). Nuclei were counterstained using Mayer's hematoxylin for 3 min and blued for 10 min in running tap water.

RT-qPCR for EBV gene expression

RNA was isolated as above and cDNA was synthesized using iScript (BioRad). Absolute quantification of the selected EBV transcripts was performed by TaqMan real time PCR (Applied Biosystems) using the AQ plasmid as the quantitative standards (3) together with gene-specific primers (Alta Bioscience, University of Birmingham) and FAM-TAMRA labeled TaqMan probes (Eurogentec), previously described (4). Endogenous control assays for Glyceraldehyde 3-phosphate dehydrogenase (*GAPDH*, assay ID hs99999905.m1 VIC) was obtained from ThermoFisher. EBV transcripts were normalized to *GAPDH* and represented as EBV mRNA/*GAPDH*.

Blocking of EPHA2

For the antibody blocking assay, AdAH cells or GC organoids were pre-incubated with an EPHA2 antibody (2 or 4 μg/ml; R&D Systems, AF3035-SP) at 4 °C for 1 h. Pre-incubated cells were exposed to EBV in the presence of indicated antibodies via transfer infection. The percentage of infected epithelial cells was determined by flow cytometry at 4 dpi. For the EPHA2 ligand ephrinA1-Fc, AdAH cells or GC organoids were pre-incubated with ephrinA1-Fc 1 or 10 μg/ml (R&D Systems, 6417-A1-050) at 37 °C for 1 h. The pretreated cells were infected with EBV via transfer infection and infection efficiency was determined by flow cytometry 4 dpi.

Cell-free virus concentration and infection

Supernatant of $1x10^6$ induced Akata cells was concentrated via centrifugation at 70,000 g at 4 °C for 2 h, the pellet was resuspended in 100μl of medium (organoid medium for organoids and AdAH medium for AdAH cells) and used for infection immediately.

3D microinjection

For microinjection, 3D organoids were seeded in 50 µL of Matrigel in 4-well multidishes (Thermo Scientific, Waltham, MA). Organoids were microinjected with induced EBV+ Akata B cells or cell-free virus on day 10 using a micromanipulator and microinjector (Eppendorf FemtoJet 4i) together with a stereomicroscope within a sterile safety cabinet (Kojair Biowizard Silverline, Vippula, Finnland). Up to 30 organoids were injected in each well. Injections were performed in a paired manner inside and outside the organoids on the same plate. 4 d after injection, organoids were removed from Matrigel and analyzed for EBV infection efficiency by flow cytometry.

SUPPLEMENTARY REFERENCES

1. Manders EMM, Verbeek FJ, Aten JA. Measurement of co-localization of objects in dual-colour confocal images. Journal of Microscopy. 1993 Mar;169(3):375–82.

2. Kayisoglu O, Weiss F, Niklas C, Pierotti I, Pompaiah M, Wallaschek N, et al. Location-specific cell identity rather than exposure to GI microbiota defines many innate immune signalling cascades in the gut epithelium. Gut. 2020 Jun 22;gutjnl-2019-319919.

3. Tierney RJ, Shannon-Lowe CD, Fitzsimmons L, Bell AI, Rowe M. Unexpected patterns of Epstein-Barr virus transcription revealed by a high throughput PCR array for absolute quantification of viral mRNA. Virology. 2015 Jan 1;474:117–30.

4. Bell AI, Groves K, Kelly GL, Croom-Carter D, Hui E, Chan ATC, et al. Analysis of Epstein–Barr virus latent gene expression in endemic Burkitt's lymphoma and nasopharyngeal carcinoma tumour cells by using quantitative real-time PCR assays. Journal of General Virology. 2006 Oct 1;87(10):2885–90.
