# Supplementary material for: Ephrin receptor A2, the epithelial receptor for Epstein-Barr virus entry, is not available for efficient infection in human gastric organoids
Source: PLoS Pathog. 2021 Feb 17;17(2):e1009210. doi: 10.1371/journal.ppat.1009210 (PMC7935236; doi:10.1371/journal.ppat.1009210)
Supplement: S1 Fig — (A) Organoid-derived monolayers were infected with cell-free virus or by transfer infection. 1 dpi, EBV infection efficiency was evaluated by flow cytometry. Bars represent means with SD of 16 experiments in organoids derived from 6 patients. (B and C) Organoids were microinjected with EBV-positive, lytically induced Akata B cells or cell-free virus at the apical or basolateral side. (B) Illustration and representative image of microinjected organoids. (C) At 4 dpi, EBV infection efficiency was evaluated by flow cytometry. Data represent means with SD from two independent experiments. (PDF) [file ppat.1009210.s002.pdf]

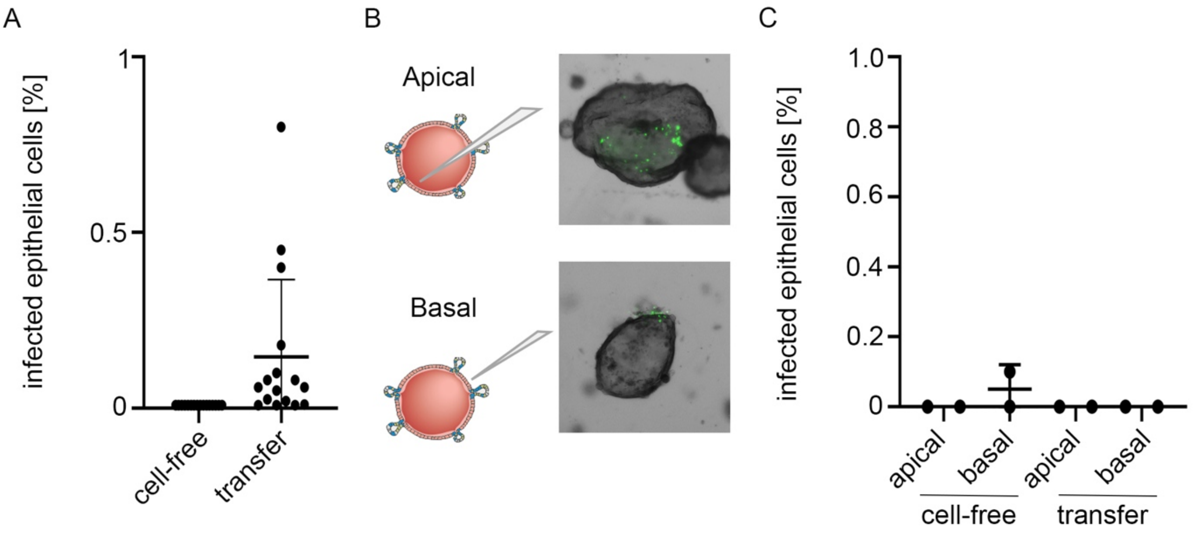

S1 Fig: EBV infects healthy gastric epithelial cells very inefficiently.
(A) Organoid-derived monolayers were infected with cell-free virus or by transfer infection. 1 dpi, EBV infection efficiency was evaluated by flow cytometry. Bars represent means with SD of 16 experiments in organoids derived from 6 patients. (B and C) Organoids were microinjected with EBV-positive, lytically induced Akata B cells or cell-free virus at the apical or basolateral side. (B) Illustration and representative image of microinjected organoids. (C) At 4 dpi, EBV infection efficiency was evaluated by flow cytometry. Data represent means with SD from two independent experiments.
