# Supplementary material for: Ephrin receptor A2, the epithelial receptor for Epstein-Barr virus entry, is not available for efficient infection in human gastric organoids
Source: PLoS Pathog. 2021 Feb 17;17(2):e1009210. doi: 10.1371/journal.ppat.1009210 (PMC7935236; doi:10.1371/journal.ppat.1009210)
Supplement: S2 Fig — At 4 dpi, EBV infection efficiency was evaluated by flow cytometry (A) and fluorescence microscopy (B). (A) Data represent means with SD from two independent experiments. (B) Representative images from two independent experiments. Scale: 400 μm. #30 and 72 refer to patient IDs. (PDF) [file ppat.1009210.s003.pdf]

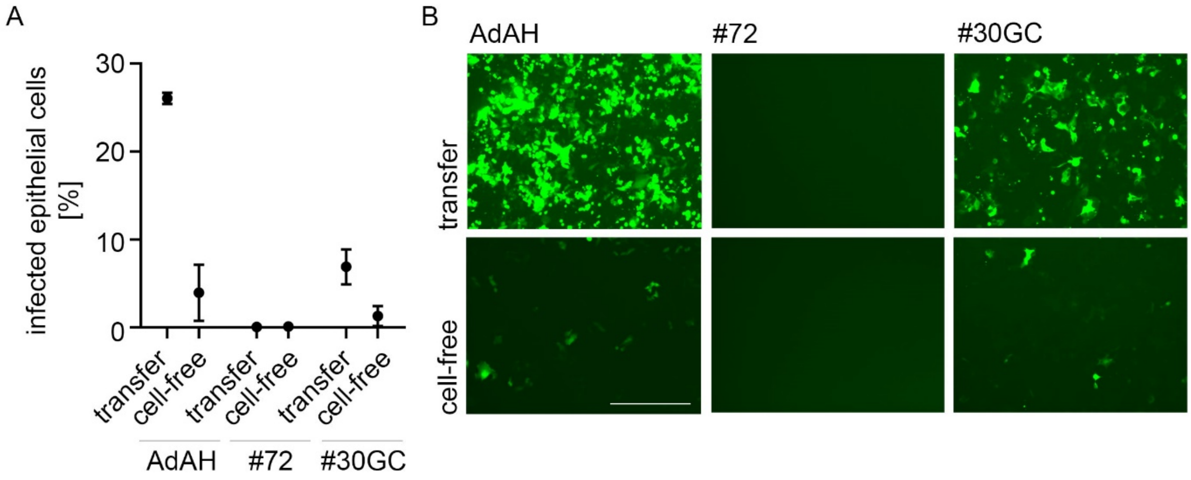

S2 Fig: B cell-mediated transfer infection more efficient than cell-free virus infection in cell lines as well as organoids.

At 4 dpi, EBV infection efficiency was evaluated by flow cytometry (A) and fluorescence microscopy (B). (A) Data represent means with SD from two independent experiments. (B) Representative images from two independent experiments. Scale: 400 µm. #30 and 72 refer to patient IDs.
