# Supplementary material for: Ephrin receptor A2, the epithelial receptor for Epstein-Barr virus entry, is not available for efficient infection in human gastric organoids
Source: PLoS Pathog. 2021 Feb 17;17(2):e1009210. doi: 10.1371/journal.ppat.1009210 (PMC7935236; doi:10.1371/journal.ppat.1009210)
Supplement: S3 Fig — (A) Immunofluorescence was performed for EPHA2 and E-cadherin. DNA was counterstained with Hoechst. Scale: 25 μm. (B) Co-localization analysis for EPHA2 and E-cadherin was performed using ImageJ. Mander’s coefficients M1 and M2 with SD were calculated from four individual images (1). (C) Scheme depicting localization of EPHA2 and E-cadherin in adherens junctions of normal human gastric organoids. AJ: adherens junction, TJ: tight junction. (D) Immunofluorescence was performed for EPHA2. DNA was counterstained with Hoechst. Scale: 25 μm. (E) Immunofluorescence was performed for EPHA2. Actin filaments were stained with Phalloidin, DNA was counterstained with Hoechst. #1, 30, 71, 72 refers to patient IDs. Scale: 25 μm. Images in A and D are identical with images shown in Fig 5C and 5D in the main manuscript. The separate display was chosen for space reasons: The main manuscript contains the overlay of EPHA2 and DNA and the supplement contains full display of separate channels. (PDF) [file ppat.1009210.s004.pdf]

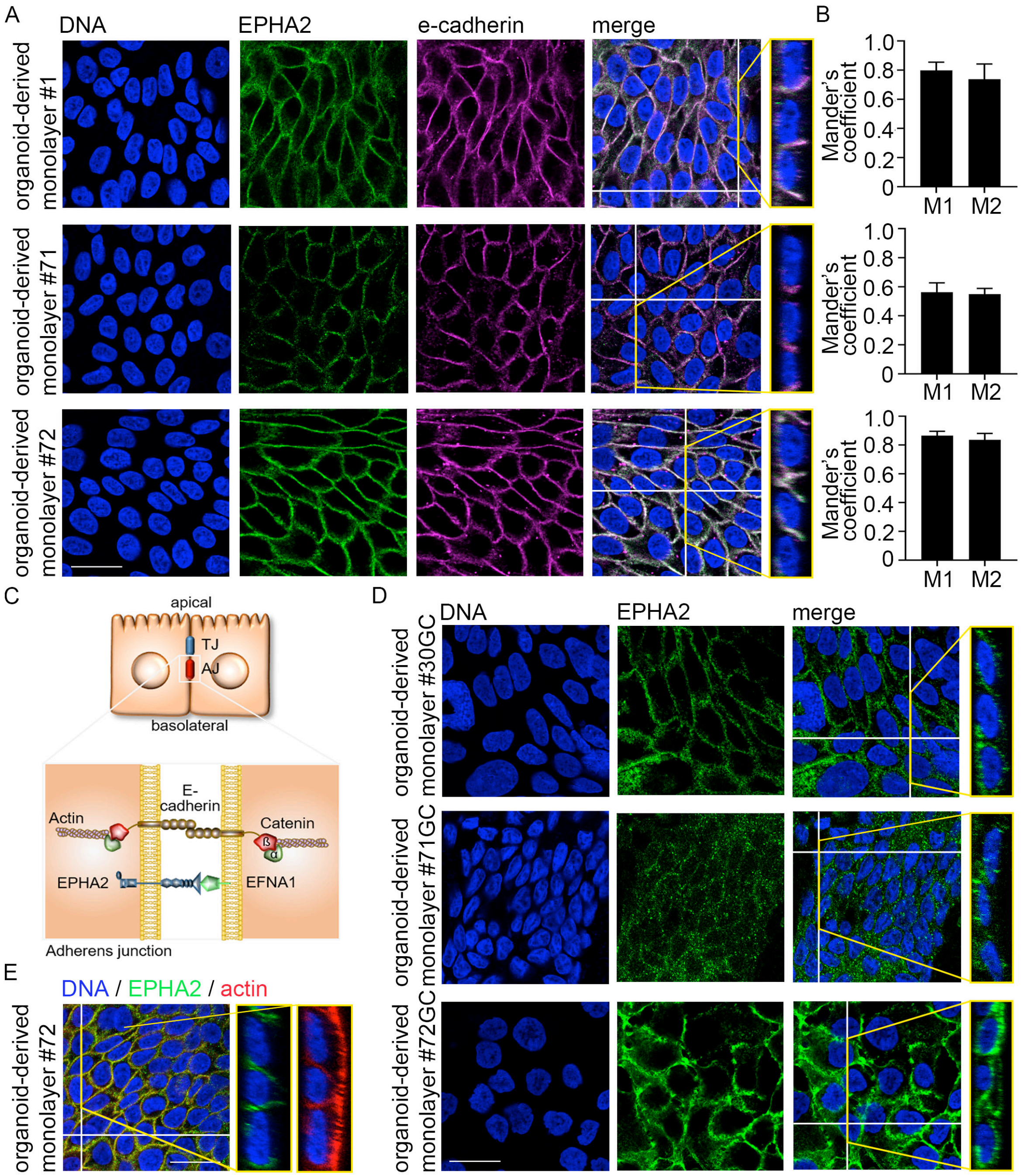

S3 Fig: In normal but not cancer organoid-derived monolayers, EPHA2 co-localizes with E-cadherin in cell-cell junctions.

(A) Immunofluorescence was performed for EPHA2 and E-cadherin. DNA was counterstained with Hoechst. Scale: 25 µm. (B) Co-localization analysis for EPHA2 and E-cadherin was performed using ImageJ. Mander's coefficients M1 and M2 with SD were calculated from four individual images (1). (C) Scheme depicting localization of EPHA2 and E-cadherin in adherens junctions of normal human gastric organoids. AJ: adherens junction, TJ: tight junction. (D) Immunofluorescence was performed for EPHA2. DNA was counterstained with Hoechst. Scale: 25 µm. (E) Immunofluorescence was performed for EPHA2. Actin filaments were stained with Phalloidin, DNA was counterstained with Hoechst. #1, 30, 71, 72 refers to patient IDs. Scale: 25 µm. Images in A and D are identical with images shown in Figure 5 C and D in the main manuscript. The separate display was chosen for space reasons: The main manuscript contains the overlay of EPHA2 and DNA and the supplement contains full display of separate channels.
