# Supplementary material for: Ephrin receptor A2, the epithelial receptor for Epstein-Barr virus entry, is not available for efficient infection in human gastric organoids
Source: PLoS Pathog. 2021 Feb 17;17(2):e1009210. doi: 10.1371/journal.ppat.1009210 (PMC7935236; doi:10.1371/journal.ppat.1009210)
Supplement: S4 Fig — (A) Immunofluorescence was performed for EPHA2 and cell-cell contact marker E-cadherin. DNA and actin filament counterstaining with Hoechst and phalloidin respectively indicate the orientation of the cells with the apical side facing the lumen of the organoid. Images were taken on a confocal microscope and the 3D reconstruction was built by LAS software (Leica). (B) Images of paraffin sections of healthy gastric mucosa or cancer tissue stained for EPHA2. Scale: (A) 20 μm, (B) 10 μm. (PDF) [file ppat.1009210.s005.pdf]

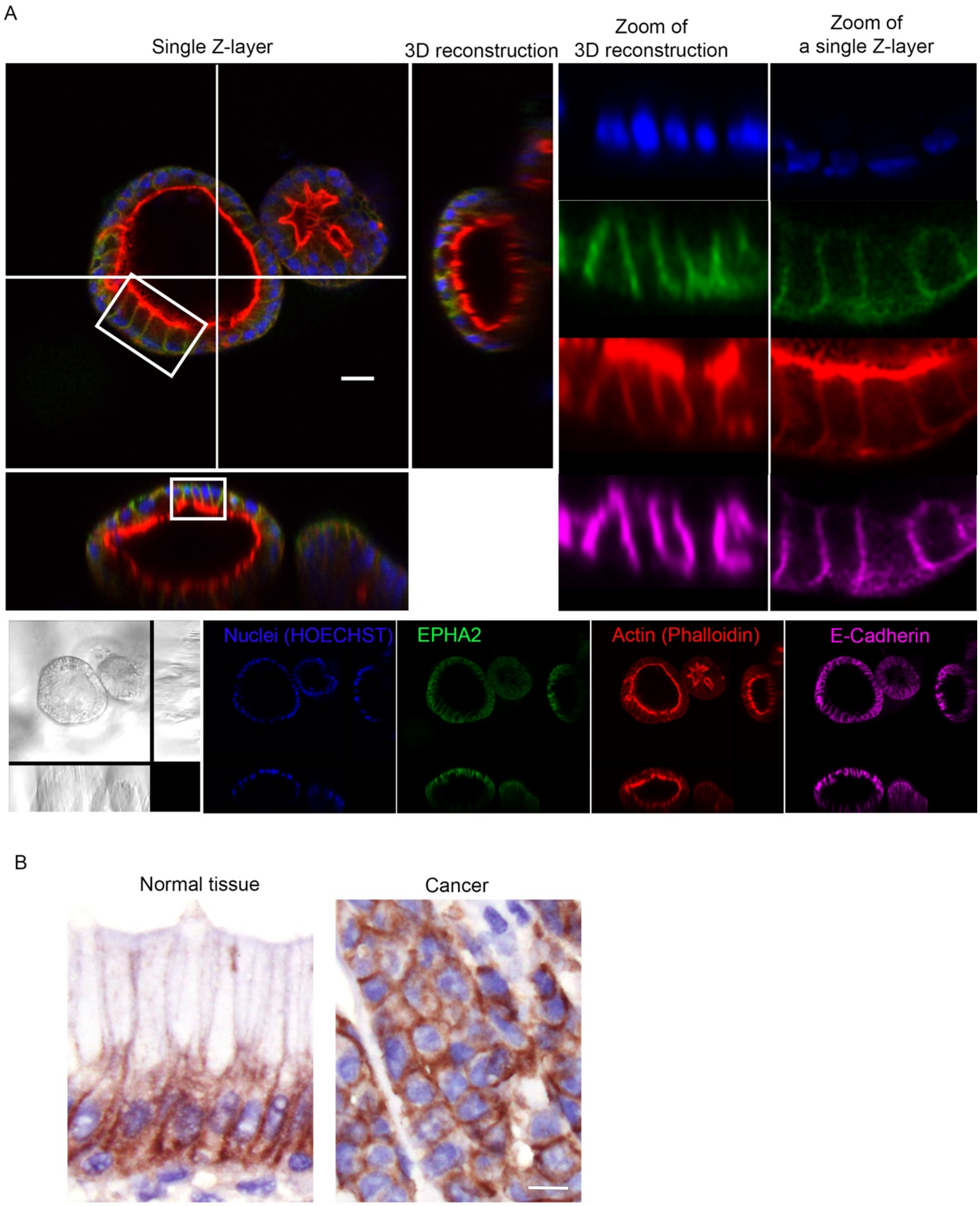

S4 Fig: EPHA2 localizes to cell-cell junctions in normal 3D organoids as well as in gastric tissue.

(A) Immunofluorescence was performed for EPHA2 and cell-cell contact marker E-cadherin. DNA and actin filament counterstaining with Hoechst and phalloidin respectively indicate the orientation of the cells with the apical side facing the lumen of the organoid. Images were taken on a confocal microscope and the 3D reconstruction was built by LAS software (Leica). (B) Images of paraffin sections of healthy gastric mucosa or cancer tissue stained for EPHA2. Scale: (A) 20 μm, (B) 10 μm
