# Supplementary material for: Ephrin receptor A2, the epithelial receptor for Epstein-Barr virus entry, is not available for efficient infection in human gastric organoids
Source: PLoS Pathog. 2021 Feb 17;17(2):e1009210. doi: 10.1371/journal.ppat.1009210 (PMC7935236; doi:10.1371/journal.ppat.1009210)
Supplement: S1 Table — (PDF) [file ppat.1009210.s006.pdf]

Supplementary table 1: Organoid medium composition for human gastric organoids. ROCK inhibitor was added only after the initial seeding and passaging of the organoids. For basal medium (AD++), Advanced Dulbecco's modified Eagle medium (DMEM)/F12 supplemented with 10 mmol/l HEPES and GlutaMAX 1 X was used. CM: conditioned medium; inh.: inhibitor; N-Ac: N-acetylcysteine; EGF: epidermal growth factor; FGF-10: fibroblast growth factor-10; TGF-β: transforming growth factor-β; ROCK: Rho-associated coiled-coil forming protein serine/threonine kinase.

**Reagent**	**Supplier**	**Catalog number**	
**Organoid maintenance**			
Matrigel	Corning	356231	
**Basal medium (AD++)**			
HEPES	Invitrogen	15630-056	
GlutaMAX-I	Invitrogen	35050-079	
Advanced DMEM/F12 (AD)	Invitrogen	12634-028	
**Medium components**			
**Reagent**	**Supplier**	**Catalog number**	**Final concentration**
AD++			30%
WNT CM	Stable cell line		50%
R-Spondin CM	Stable cell line		10%
Noggin CM	Stable cell line		10%
Primocin	Invivogen	Ant-pm-1	100 µg/ml
B27	Invitrogen	12587-010	1 X
N-Ac	Sigma-Aldrich	A9165-5G	1.25 mM
EGF	Peprotech	AF-100-15	50 ng/ml
FGF-10	Peprotech	100-26	100 ng/ml
Gastrin-I	Tocris	3006	1 nM
TGF-β inh. (A-83-01)	Tocris	2939	2 µM
ROCK-inh (Y-27632)	AbMole Bioscience	M1817	10 µM
