# Supplementary material for: Ephrin receptor A2, the epithelial receptor for Epstein-Barr virus entry, is not available for efficient infection in human gastric organoids
Source: PLoS Pathog. 2021 Feb 17;17(2):e1009210. doi: 10.1371/journal.ppat.1009210 (PMC7935236; doi:10.1371/journal.ppat.1009210)
Supplement: S2 Table — ROCK inhibitor was added only after the initial seeding and passaging of the organoids. For basal medium (AD++), Advanced Dulbecco’s modified Eagle medium (DMEM)/F12 supplemented with 10 mmol/l HEPES and GlutaMAX 1 X was used. CM: conditioned medium; inh.: inhibitor; N-Ac: N-acetylcysteine; EGF: epidermal growth factor; FGF-10: fibroblast growth factor-10; TGF-β: transforming growth factor-β; ROCK: Rho-associated coiled-coil forming protein serine/threonine kinase. (PDF) [file ppat.1009210.s007.pdf]

Supplementary table 2: Patient information for the organoid lines used in this study.

Patient #	Gender	Age	Surgery
1	F	32	Sleeve stomach
30GC	M	76	Stomach carcinoma
32	F	47	Sleeve stomach
36	M	55	Stomach carcinoma
42	M	58	Stomach carcinoma
53	M	49	Stomach carcinoma
58	F	58	Stomach carcinoma
60	M	57	Stomach carcinoma
61	F	71	Stomach carcinoma
71	F	82	Stomach carcinoma
71GC	F	82	Stomach carcinoma
72	M	79	Stomach carcinoma
72GC	M	79	Stomach carcinoma
