# Supplementary material for: Ephrin receptor A2, the epithelial receptor for Epstein-Barr virus entry, is not available for efficient infection in human gastric organoids
Source: PLoS Pathog. 2021 Feb 17;17(2):e1009210. doi: 10.1371/journal.ppat.1009210 (PMC7935236; doi:10.1371/journal.ppat.1009210)
Supplement: S3 Table — (PDF) [file ppat.1009210.s008.pdf]

Supplementary table 3: Primer sequences.

Name	Sequence (5'-3')	Method
hEPHA2_F	AAGGAAGTGGTACTGCTGGA	qPCR
hEPHA2_R	ACGTTGCACACGGAGTACAT	qPCR
hGAPDH_F	GTTTCTATAAATTGAGCCCGC	qPCR
hGAPDH_R	TGTAAACCATGTAGTTGAGGT	qPCR
hEPHA2cloning_SpeI_F	TAATAAACTAGTATGGAGCTCCAGGC AGCCCG	Cloning lentiviral *EPHA2* overexpression plasmid
hEPHA2cloning_XhoI_R	TTATTACTCGAGCTAGATGGGGATCC CCACAGTG	Cloning lentiviral *EPHA2* overexpression plasmid
ApoB_F	TGAAGGTGGAGGACATTCCTCTA	PCR
ApoB_R	CTGGAATTGCGATTTCTGGTAA	PCR
EBER2_F	CCCTAGTGGTTTCGGACACA	PCR
EBER2_R	ACTTGCAAATGCTCTAGGCG	PCR
EBNA1_F	GTCATCATCATCCGGGTCTC	PCR
EBNA1_R	TTCGGGTTGGAACCTCCTTG	PCR
gp220_F	GGCTGGTGTCACCTGTGTTA	PCR
gp220_R	CCTTAGGAGGAACAAGTCCC	PCR
LMP1_F	CAGTCAGGCAAGCCTATGA	PCR
LMP1_R	CTGGTTCCGGTGGAGATGA	PCR
