# Supplementary figures and images for: Ephrin receptor A2, the epithelial receptor for Epstein-Barr virus entry, is not available for efficient infection in human gastric organoids
Source: PLoS Pathog. 2021 Feb 17;17(2):e1009210. doi: 10.1371/journal.ppat.1009210 (PMC7935236; doi:10.1371/journal.ppat.1009210)

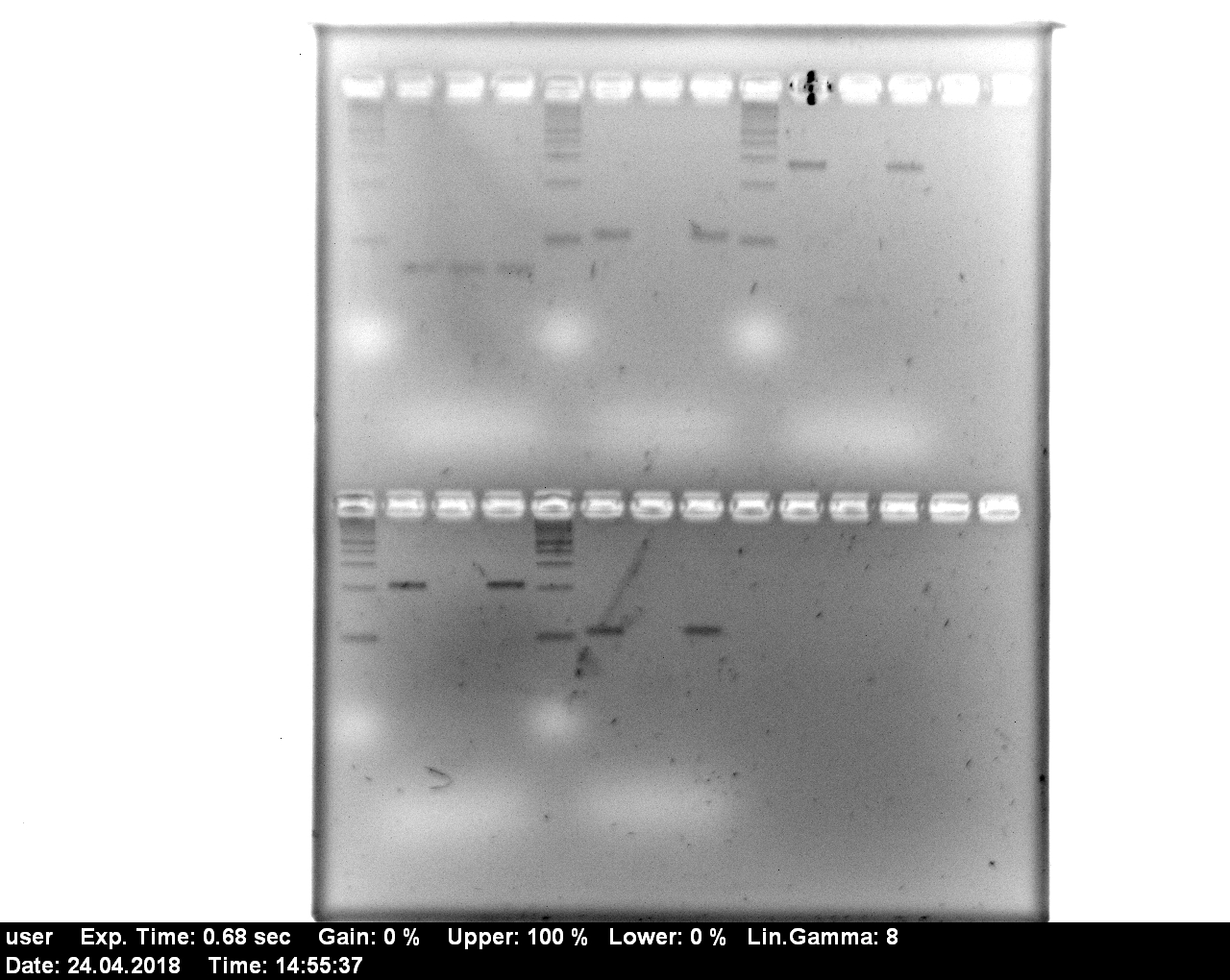

Supplement: S2 Data — (JPG) [file ppat.1009210.s010.jpg]
